# Voices from the field: exploring practitioners' experiences and perceptions of the voices of athletes (VOA) in the pacific islands

**DOI:** 10.3389/fspor.2024.1351451

**Published:** 2024-02-27

**Authors:** Hee Jung Hong, Brian Minikin

**Affiliations:** Faculty of Health Sciences and Sport, University of Stirling, Stirling, United Kingdom

**Keywords:** athlete empowerment, educational initiatives, holistic development, sport education, transferrable skills development

## Abstract

This exploratory study aims to gain insights into practitioners' experiences and perceptions of the “Voices of Athletes” (VOA), a specialized athlete support program, developed and implemented in the Pacific Islands. Semi-structured interviews enabled participants to share detailed experiences, with 14 practitioners participating. Thematic analysis of the data identified five key themes: “Fostering Athlete Empowerment”, “Impact of Sport on Education and Social Change”, “Expanding VOA's Reach and Impact”, “VOA's Role in Preparing Athletes for Post-Sport Life”, and “Optimizing VOA Implementation and Experience”. The findings demonstrated that the VOA plays a critical role in empowering athletes and assisting them in becoming leaders within their societies. Practitioners emphasized the power of sport as an effective channel for education and inspiration, and the potential for the VOA framework to be applied in various contexts and regions. The study also revealed that the VOA helps athletes prepare for life after sport, contributing to increased self-esteem, development of transferable skills, and awareness of their social roles. The findings also emphasized the need for VOA improvements, including enhanced interactivity, larger spaces, and financial support. Recognition from sport governing bodies and coaches could broaden the program's reach and impact. Incorporating internship schemes within the VOA or related programs could address life after sport more effectively. Developing written VOA guidelines would ensure consistent, sustainable delivery, supporting its potential for wider implementation and adaptation, contributing to holistic development for athletes and young people globally.

## 1 Introduction

High-performance sports systems worldwide commit substantial resources, including finances, personnel, and time, to support exceptionally promising athletes. A growing trend has emerged among nations to increase investment in their elite sports systems over recent decades (1). This shift can be partly attributed to the understanding that success on the international stage brings prestige, national pride, and economic benefits (2, 3). Achieving international success in sports depends on athletes engaging in intensive training and participating in a number of competitions over an extended period, requiring significant individual investments (4, 5). Financial investments in training, equipment, and travel for competitions can place a considerable burden on athletes and their families (6).

Evidence suggests that providing targeted support programs for athletes, such as access to specialized coaching, sport science services, and financial assistance, can significantly enhance their performance and chances of success (7–9). Such athlete support programs not only reduce the financial burden on athletes and their families but also establish an environment conducive to optimizing their potential and achieving their athletic goals. By understanding and addressing the unique needs and challenges faced by individual athletes, these programs can contribute to the overall development and success of high-performance sports systems (10–12).

Athletes, along with their families and sports clubs, can only invest limited resources, which might hinder their chances of success. In this respect, athlete support programs are designed to offer supplementary resources to selected athletes, thus subsidizing their endeavors. These programs identify the most promising athletes and engage them in a sequence of interventions focused on improving their performance, eventually increasing their probability of success (13). Previous studies have emphasized the importance of holistic and multifaceted support for athletes. For instance, Ryba et al. (14) highlighted the value of considering the cultural context and mental well-being of athletes in support programs, suggesting that tailored interventions can enhance performance outcomes.

Talent development environments should be also structured to optimize athletes' long-term development, including their physical, psychological, and social competencies (15). Athlete support programs that encompass various forms of support, such as financial, educational, and mentoring, have been shown to be more effective in enhancing athletes' performance (16). Comprehensive support systems may also help athletes develop key life skills and transferrable competencies that contribute to their overall well-being and success beyond their athletic careers (17). In this regard, athlete support programs play a crucial role in providing additional resources to aspiring athletes, increasing their chances of success. Thus, comprehensive, multifaceted, and context-specific support programs are ciritical for optimizing athletes' performance and overall development.

Initiatives worldwide have been developed to assist athletes with career development and transitions beyond sport (8, 11, 18). Athletes face a number of challenges during and after their athletic careers (15, 19), prompting experts to call for sport governing bodies, organizations, and National Olympic Committees (NOCs) to support athletes in managing both athletic and non-athletic careers, ensuring their overall well-being. As a result, sport governing bodies have established athlete support programs to assist athletes in navigating their sports careers and preparing for life after sport (8, 20, 21). Research on organizational support in high-performance sport is underdeveloped compared to studies on social support for such athletes (22, 23).

Previous research has shed light on organizational support's different dimensions, with some studies examining the role of organizational support in athlete burnout (24), mental health (17), and well-being (25). In addition, research has explored how organizational support contributes to athletes' satisfaction (26), development (27), and overall career development and transition (8, 11, 15). However, despite the growing interest in organizational support in high-performance sports, the literature still has gaps. For example, researchers have called for more research on organizational support in different cultural contexts and among various sports (28). Researchers have also highlighted the need to better understand the specific mechanisms by which organizational support influences athletes' outcomes and experiences (29).

The majority of studies focusing on athletes' career development and transitions have been carried out in Australia, Europe, and North America (30). Park et al. (19) also highlighted that a significant portion of research on athlete support systems originated in Western countries, with 10 instances in Australia, 45 in Europe, and 60 in North America. To counteract this geographical unevenness, Hong and Coffee (8) explored athlete support programs in 19 countries spanning Africa, Asia, Europe, North America, South America, and Oceania. Despite their work presenting a comprehensive assessment of organizational support through athlete support programs across multiple continents, the nations examined were primarily developed, such as Australia and New Zealand within the Oceania region. This leads to an evident gap in research concerning athlete support programs in developing nations, particularly in the Pacific Islands, excluding Australia and New Zealand. The lack of representation in these areas constrains the generalizability of study outcomes and obstructs the formulation of efficient support approaches tailored to the distinct requirements and conditions of athletes from these regions. Expanding research to include Pacific Island developing countries would offer not only a broader understanding of the global scope of athlete support systems but also reveal potential cultural, social, and economic factors influencing the success of organizational support via structured programs.

To the authors' understanding, there are no structured athlete support programs in the Pacific Island regions equivalent to those established in other nations for assisting athletes with career growth and transitions. Nonetheless, by 2006, the Oceania National Olympic Committees (ONOC) had set up its regional sports education initiatives, collectively referred to as the Oceania Sport Education Program (OSEP). Initially centered on educating coaches and sports administrators, OSEP also facilitated the emergence of new initiatives like Voices of Athletes (VOA). VOA's inception was the result of collaboration between two athlete-focused education programs introduced to Oceania: a drug education program by the Oceania Regional Anti-Doping Organisation (ORADO) and the Stop HIV initiative, co-funded by the United Nations Programme on HIV/AIDS (UNAIDS), the South Pacific Commission (SPC), and ONOC. Launched in 2004 as a pilot program by the World Anti-Doping Association (WADA) and ONOC, these initiatives joined forces in 2007, creating a platform to deliver messages directly to athletes. The first effort to involve athletes began in 2007 during the Pacific Games in Samoa, where an education booth was set up in the Games Village (31). This effort led to several athlete outreach activities at various national and regional sports events and within specific countries. These endeavors successfully increased awareness and allowed athletes to discuss different issues impacting their lives.

Although VOA represents progress in athlete support in the Pacific Islands, more research and program expansion are required to address the region's athletes' unique challenges and needs. While examining athletes' perspectives of athlete support programs is crucial, and indeed forms part of a larger project that encompasses the present study, there is an increasing awareness of the value in exploring practitioners' experiences and perceptions of these programs. Gaining insights from practitioners can contribute to enhancing the effectiveness and quality of such initiatives, as they possess unique knowledge and understanding of the program's workings and impact on athletes. Hong and Coffee (8) explored who is responsible for delivering athlete support programs and how sport organizations support practitioners in developing their competence to assist elite athletes. They suggested that future research should investigate practitioners' views on the content and delivery of such programs to enhance their impact. Considering this evidence, it is crucial to extend the focus beyond athletes' experiences and incorporate the perspectives of practitioners involved in the VOA to better understand its functioning and potential areas of improvement. By investigating practitioners' experiences and perceptions of VOA and similar initiatives, researchers can contribute to developing comprehensive and culturally sensitive support strategies that promote the holistic development and well-being of Pacific Island athletes and beyond.

The VOA emerged from the need for coordination and collaboration among multiple agencies in resource-limited settings (32). It serves as an educational platform supporting athletes’ personal growth, empowering well-rounded individuals positively impacting communities and sport. Continually adapting and expanding its initiatives, the VOA demonstrates the potential for similar programs to address diverse athlete needs and foster holistic development. VOA's primary objective is to inspire athletes to become positive role models, captured by the motto “Be a Leader”. The program comprises four distinct initiatives, each addressing different aspects of athlete development and responsibility (31, 32). **Play True** adapted from WADA's original anti-doping programs, promotes a culture of clean sport and integrity among athletes in the Pacific Region. **Stay Healthy**, which originated from Stop HIV initiatives, now covers broader health and lifestyle issues, encouraging healthier lifestyles. **Go Green**, adopted from IOC-established environmental initiatives, uses sport to spread conservation messages and urges athletes to be environmentally responsible. **Play Safe** addresses athletes' concerns about harassment and abuse, focusing on protection, especially for those vulnerable to authority figure abuse.

The various practitioners deliver these initiatives, motivating athletes to engage in each component and helping them absorb key messages from each segment. The present study, thus, aims to examine practitioners' experiences and perceptions of VOA, as they interact directly with athletes. In doing so, this study seeks to provide insights into an athlete support program in the Pacific Island region, an area less extensively explored compared to athlete support programs in other countries documented in previous research. Gaining insights from practitioners involved in athlete support programs in this region will not only inform the ongoing development and implementation of the program but also contribute to the broader understanding of athlete support systems in diverse contexts. This valuable information can help sport governing bodies develop well-established support systems and initiatives tailored to various settings.

### 1.1 Theoretical background

Communities of practice (CoP), a concept introduced by Wenger (33), are prevalent in various aspects of our lives, which includes social learning processes that occur within groups sharing common interests, goals, and practices, with participation levels ranging from core membership to more peripheral involvement. Members within a CoP are informally connected through their collaborative activities, mutual engagement, and shared learning experiences. Characterized by mutual engagement, a shared repertoire of resources, and a joint enterprise, CoPs enable members to learn from one another, develop shared understandings, and generate new knowledge through interactions and collaborations (33). Distinct from communities of interest or geographic communities, CoPs need a shared practice. As CoPs evolve, they progress through various stages of development, characterized by differing levels of interaction among members and distinct types of activities. These communities form around topics of importance to their members, with their practices reflecting the members' understanding of what matters most. Although external factors can influence this understanding, CoPs create practices that serve as their response to these external influences. As a result, even when a community's actions align with an external mandate, it is the community itself that shapes the practice. Thus, CoPs can be perceived as fundamentally self-organizing systems, capable of adapting and evolving based on the needs and interests of their members (33).

This social learning approach has significant implications for understanding the dynamics of various professional contexts. Willem et al. (34) examined the difficulties that sport organizations and policymakers across the world faced in implementing Sport-for-All. This field is both practical and rich in knowledge, involving a wide network of knowledge creation and sharing among various groups, including multiple agencies, professionals, and volunteers. Using the concept of CoP as their theoretical framework, Willem et al. (34) investigated the role of governing bodies of sport as facilitators of knowledge exchange within Sport-for-All communities. The findings demonstrated that governing bodies facilitate knowledge sharing, new ideas exploration, and knowledge creation in Sport-for-All communities. However, these bodies were not fully utilizing online tools strategically. The authors recommended a more strategic approach to new media tools usage, allowing CoP standards to emerge naturally instead of determining them. In a broader context of sport, Culver and Trudel (35) sought to clarify the concept of CoP by exploring learning opportunities outside conventional classroom environments. These opportunities, known as workplace learning, non-formal learning, informal learning, or incidental learning, are marked by the crucial role peers play in the learning process (35). The authors investigated the potential of CoPs in sports by offering a concise overview and comparison of the concept, as well as reviewing recent research in the area. They highlighted the importance of adhering to Wenger's (33) framework when applying CoPs in sports. Culver and Trudel's study contributed to fostering a better understanding of CoPs in sports while promoting further discussions on the potential applications of this concept in the fields of coaching education and sports management.

In applying the CoP framework to the present study, which explores practitioners' experiences and perceptions of the VOA in the Pacific Island region, valuable insights can be gained regarding the mechanisms through which CoPs facilitate learning, collaboration, and the dissemination of best practices among VOA practitioners. This understanding can enhance the effectiveness and impact of the athlete support program such as the VOA, furthering the field's knowledge and practical applications. By examining the VOA from the CoP perspective, the study can explore the relationships and interactions among practitioners, how they engage in shared activities, and how they collectively deliver the existing resources to achieve the program's objectives. This can provide a richer understanding of the factors that influence practitioners' perceptions and experiences, as well as shed light on the challenges they face in delivering the VOA and the strategies they employ to overcome these challenges. In addition, the application of the CoP can help identify areas where VOA practitioners can further enhance their collaborative learning and knowledge sharing processes. Thus, the application of the CoP to the present study of the VOA in the Pacific Island region has the potential to provide a deeper understanding of practitioners' experiences and perceptions, as well as uncover opportunities for enhancing collaboration, learning, and the sharing of best practices within the program. This, in turn, can contribute to the ongoing development and effectiveness of the VOA and similar athlete support initiatives in different contexts.

## 2 Materials and methods

### 2.1 Design

This study employed an intrinsic case study design to explore in-depth insight into the experiences of VOA practitioners in implementing the VOA. An intrinsic case study is regarded as a research approach focused on understanding a specific phenomenon, which researchers need to clearly identify and define what makes such phenomenon unique and stands out from others (36). Our research design facilitated a detailed exploration of a specific case, focusing on the personal experiences of those involved. This was to gain a deep understanding of the participants' perspectives on their experiences, making an interpretive phenomenological approach suitable. This approach is based on an interpretivist paradigm, aligning with relativist ontology and subjectivist epistemology (37). This paradigm enabled us to explore the individual perspectives and interpretation of experiences (37, 38). Interpretive phenomenology aims to describe, comprehend, and interpret phenomena, thereby uncovering the essence of lived experiences (39, 40). To thoroughly capture the richness of the participants' experiences, we employed semi-structured interviews.

### 2.2 Participants

To explore the practitioners' perception and experience of delivering the VOA, a total of 14 practitioners participated in the study. The roles of the practitioners included VOA Champions (*n* = 10; both former and active athletes), chair of the Oceania National Olympic Committee (ONOC) Athletes Commission (*n* = 1), coordinator of the VOA (*n* = 1), creator and developer of the VOA (*n* = 1), and VOA project officer (*n* = 1). They are from five different countries including American Samoa, Fiji, Samoa, Papua New Guinea, and Vanuatu. Eight of them are female and the rest were male (*n* = 6; see Table 1).

**Table 1:** Participants.

Participants	Gender	Nationality	Role
Practitioner 1	Male	American Samoa	VOA champion (Former athlete)
Practitioner 2	Male	Fiji	VOA champion (Active athlete)
Practitioner 3	Female	Fiji	VOA champion (Former athlete)
Practitioner 4	Female	Papua New Guinea	Senior management member in the ONOC athletes commission
Practitioner 5	Female	Vanuatu	VOA champion (Active athlete)
Practitioner 6	Female	Vanuatu	VOA champion (Active athlete)
Practitioner 7	Male	Fiji	Co-ordinator
Practitioner 8	Female	Samoa	One of the key figures involved in the development and creation of the VOA
Practitioner 9	Male	PNG	VOA champion (Active athlete)
Practitioner 10	Male	Fiji	VOA champion (Former athlete)
Practitioner 11	Female	Fiji	VOA champion (Former athlete), events and travel officer for the ONOC
Practitioner 12	Female	Fiji	VOA project officer
Practitioner 13	Female	Samoa	VOA champion (Former athlete)
Practitioner 14	Male	Samoa	VOA champion (Former athlete)

### 2.3 Data collection

Semi-structured interviews were applied to gain in-depth insight into the VOA from the practitioners. An interview guide was developed based on the literature review [e.g., (8, 15, 19, 41)] and research questions and applied to each interview to ensure consistency throughout the interviews (42). The interviews were conducted to address the following: (a) role and responsibility (i.e., can you describe your role and responsibilities within the VOA?); (b) overall experience of delivering the VOA (i.e., how would you describe your overall experience in delivering the VOA?); (c) highlights/key impact factors of the VOA (i.e., what do you consider to be the most significant highlights or key impact factors of the VOA?); (d) expected/experienced outcomes of the VOA (i.e., what were the expected or experienced outcomes of the VOA for the athletes involved?); and (e) areas for improvement (i.e., in your opinion, what areas of the VOA could be improved or enhanced to better support the athletes?). Six out of 14 participants were interviewed as a pair upon their request. Interviews lasted for between 34 and 96 min (*M *= 62.00, SD = 20.63). All interviews were audio-recorded with each participant's permission.

### 2.4 Data analysis and rigor

**Figure 1:**
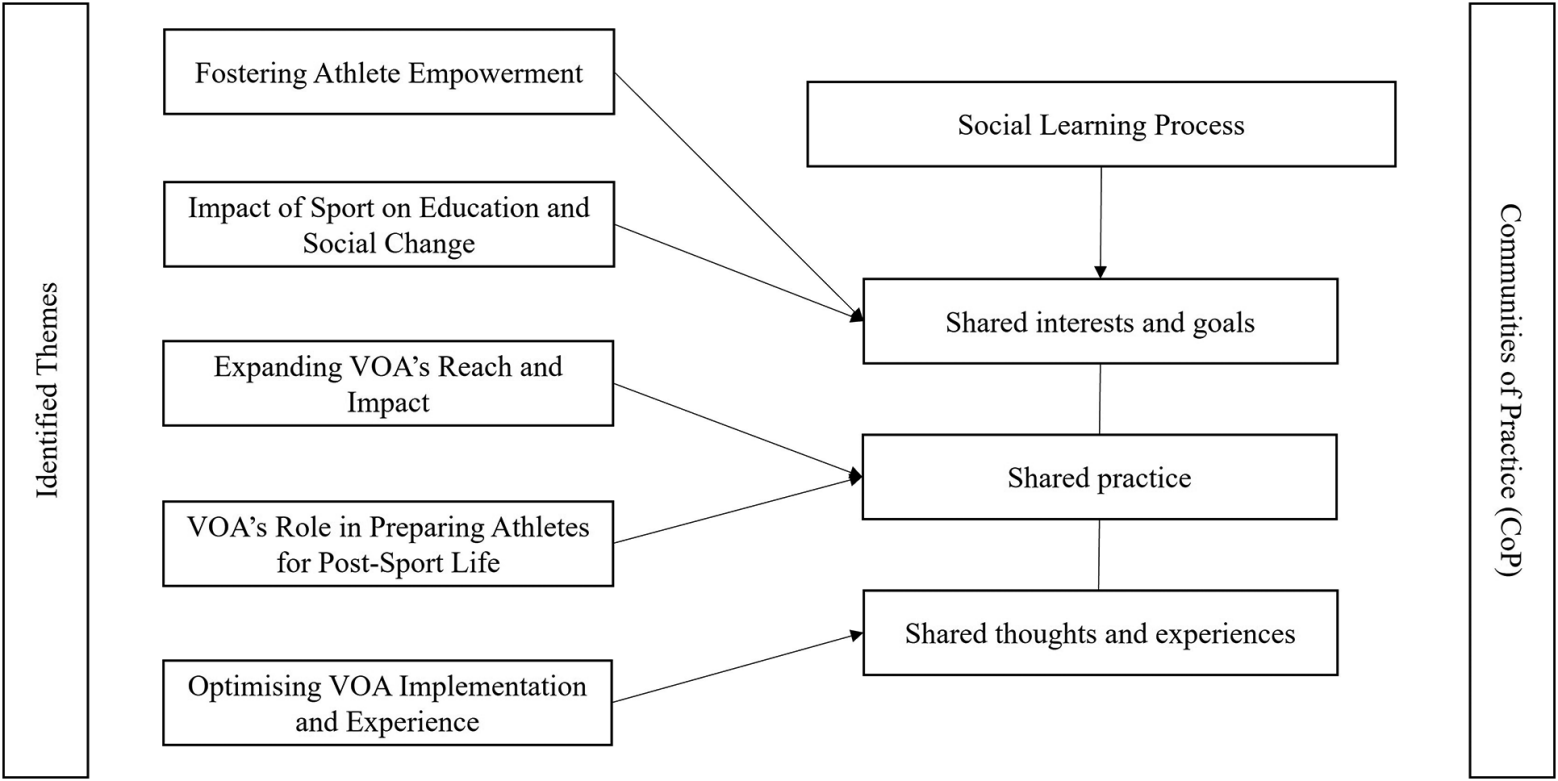
Final thema.ic map of the experiences and perceptions of the VOA practitioners.

Thematic analysis was employed to examine the transcribed data, a method that enables researchers to identify patterns within qualitative data such interview data that the present study used (43). The author adhered to the six-phase process outlined by Braun and Clarke (44). To ensure a rigorous and systematic thematic analysis, the author utilized a checklist to guide the data processing (43). Multiple readings of each transcript were conducted to achieve familiarity with the participants' narratives. Initial codes were identified and subsequently categorized into initial themes. These themes were then reviewed and refined (44), with input from two experienced qualitative researchers serving as critical friends (45). To establish trustworthiness in the data analysis process, the following measure were employed: (a) prolonged engagement with data (e.g., the lead author conducting all interviews and reading the transcripts multiple times), (b) documented theoretical and reflective thoughts, (c) documented reflections on potential codes/themes, (d) storage of raw data in well-organized archives, (e) diagrammatic representation of theme connections (see Figure 1), (f) themes and subthemes reviewed by other researchers (e.g., critical friends), (g) documentation of theme naming (46).

## 3 Results

Five key themes were identified: (a) Fostering athlete empowerment, (b) Impact of sport on education and social change, (c) Expanding VOA's reach and impact, (d) VOA's role in preparing athletes for post-sport life, and (e) Optimizing VOA implementation and experience.

### 3.1 Fostering athlete empowerment

All practitioners highlighted the critical role of the VOA in empowering athletes and assisting them in becoming leaders within their communities. The VOA provides athletes with opportunities to develop knowledge about contemporary social issues, broadening their perspectives on their societies and the world, while increasing their awareness of their potential contributions as athletes and leaders. This aligns with the overarching aim of the VOA: *Be a leader*. Practitioner 8 emphasized the core purpose of the VOA in this context, stating, “the overarching intended outcome of Voices of the Athletes was to help create athlete leaders”.

The transformative power of the VOA was further evidenced by the experiences of ten out of 14 practitioners who were VOA Champions. These practitioners, either former or active athletes themselves, had experienced the VOA as athletes before transitioning to the role of facilitator as VOA Champions. Their experiences serve as a testament to the positive impact the VOA has had on athletes, empowering them based on the knowledge and insights they gained through the program. For instance, Practitioner 10 explained, “you are not only a national hero on the court, remember off the court too you are a public figure. […] so, discipline needs to be maintained at all levels, on and off court”. This statement highlights the holistic approach of the VOA, emphasizing the importance of both athletic performance and personal conduct in shaping athletes as role models in their communities.

Practitioner 6's experience further illustrates the VOA's impact on personal growth and leadership development. Having dropped out of education at a young age due to a lack of family support, she later became a national team athlete through determination. The VOA played a crucial role in motivating her to become involved in spreading the program's messages and embracing her role as a leader within the athletic community. Participants' insights highlight the importance of the VOA in cultivating leadership qualities among athletes, empowering them to become role models and agents of change within their communities. The program's success in providing athletes with the tools and knowledge necessary to navigate contemporary social issues and develop leadership skills is evident in the experiences shared by the practitioners. The transformative effect of the VOA on the lives of these individuals, as well as the potential for similar impacts on other athletes, highlights the value of the program and its potential for fostering positive change at both individual and community levels.

### 3.2 Impact of sport on education and social change

The practitioners notably emphasized the power of sport as an effective and influential medium for educating and inspiring individuals to become aware of public health and social issues, prompting them to take action. They also highlighted the role that sport, and athletes play in leading positive changes within the Pacific Islands. Practitioner 9 remarked, “the VOA really demonstrates the power of sports because you see these national athletes here, they’re not just athletes coming, they are representatives of their country”. Many of them further elaborated on why sport is a valuable tool for educating not only athletes but also wider populations. Practitioner 10 explained, “Sport is the only tool or is the only thing that does not discriminate against anyone, regardless of your age, gender, religion, abilities, or disabilities […] So, sport is a very effective means of delivering sessions back home”.

Drawing from their appreciation for the power of sport, practitioners acknowledged the significant role the VOA plays in showcasing how sport and athletes can influence people across different societies within the Pacific Islands. However, participants expressed concern that sport is not valued as highly as it should be in many countries and that investment in sport is not yet sufficiently encouraged, despite growing recognition of its importance. This lack of investment has impacted the delivery of the VOA in various regions, limiting its reach to a wider population at different events beyond the Mini Pacific Games due to budget constraints. The practitioners' insights underline the transformative potential of sport as a vehicle for education and social change. By harnessing this potential, the VOA and similar programs can continue to promote awareness of public health and social issues, ultimately empowering individuals and fostering leadership in communities across the Pacific Islands.

### 3.3 Expanding VOA's reach and impact

One of the primary strengths of the VOA is its adaptability. Participants identified the VOA as a multifaceted framework that can be applied in various contexts and regions. They also discussed examples of programs informed by the VOA that have been implemented in their countries, demonstrating its potential for delivery outside of the Pacific Islands. Among these programs, the Hero program in Papua New Guinea was most frequently mentioned. This program, delivered by national sport heroes in remote areas where young children have limited access to education, has gained recognition and support through funding from a private company and the National Olympic Committee.

Participants specifically mentioned the potential for VOA's implementation in South American and African countries. As the VOA has functioned as a framework for other programs, as exemplified by the Hero program, this could be a future pathway for the initiative. Practitioner 4 highlighted the potential for sharing best practices not only from developed countries (Global North) to developing countries (Global South) but also in the reverse direction. This emphasizes the potential for cross-cultural learning and the adaptability of the VOA as a framework that can be tailored to suit diverse contexts, ultimately expanding its reach and impact in promoting education and social change through sport.

### 3.4 VOA's role in preparing athletes for post-sport life

Participants discussed the importance of the VOA in helping athletes prepare for their lives after sport, as the program provides opportunities to increase self-esteem and confidence, develop transferable skills (e.g., public speaking, leadership, communications skills, organizational skills), and raise awareness of their social roles as athletes. Practitioner 9, who has actively influenced children at various schools in his country using the knowledge he gained from the VOA, stated, “I have a social responsibility to make use of sports as a tool to give back to the people”. Participants expressed frustration at witnessing retired athletes returning to their everyday lives, engaging in activities they were not passionate about due to a lack of information and support. This highlights the need for programs like VOA to extend their reach and create a long-lasting impact on athletes' lives, not only during their athletic careers but also after retirement.

A challenge identified by some participants, which impedes the VOA's capacity to support athletes in developing transferable skills and preparing for their post-athletic life, was the limited awareness and accessibility of the VOA or other associated programs across various regions, primarily due to budget restrictions. Increased funding and partnerships with local organizations could help expand the reach of VOA and similar programs, making them more accessible to athletes across various regions. Practitioner 8 highlighted the difficulty athletes face in maintaining a balance between education and sports as they prepare for life after sport: “Sports people who have given up school or left school to pursue a career in sports often struggle to advance to different levels in their sport. Only a handful of sportspeople manage to pursue both, a dual career”. This observation highlights the importance of programs like the VOA in offering resources and support for athletes to navigate both their athletic and educational endeavors. It also signifies the need of promoting dual career awareness among athletes to emphasize its significance.

Recognizing the importance of preparing for life after sport, participants argued that this issue should be addressed by various stakeholders, including sports organizations, educational institutions, and community leaders. They suggested that promoting the VOA more extensively to athletes could foster greater awareness of available resources and support networks for athletes transitioning out of their sports careers. Participants also proposed the implementation of internship schemes for athletes to tackle the challenges of transitioning from sport to other careers, as such initiatives have not yet been included in the VOA or other related programs. These internships could help athletes gain valuable work experience, develop new skills, and establish professional networks, easing their transition into the workforce in the end.

### 3.5 Optimizing VOA implementation and experience

The practitioners offered valuable insights into areas for improvement in order to optimize the implementation and experience of the VOA. Common concerns included the need for more interactive activities to engage participants, larger spaces, sufficient time for athletes to complete all stations, and optimal booth locations. Regarding interactive activities, participants with extensive experience in delivering the VOA noted significant improvements over the years, as they recognized that athletes prefer learning key messages while having fun. As a result, the coordinator and facilitators addressed athletes' feedback, requesting more interactive and enjoyable activities. Despite their efforts, facilitators believe they still need to be more creative in enhancing each activity.

Many practitioners also discussed the need for support, particularly financial, to improve the program's delivery: “Money helps” (Participant 4). Financial assistance is crucial not only for the VOA's implementation but also for training more VOA Champions across the Pacific Islands, as most of them currently come from a few countries like Fiji, Papua New Guinea, Samoa, and Vanuatu. Practitioners acknowledged the power of word of mouth in promoting the VOA. While word of mouth is effective, practitioners argued that their efforts should be more widely recognized by sport governing bodies, enabling the VOA to be delivered more frequently in various regions. They also stressed the importance of gaining recognition from coaches who can encourage athletes to participate in the VOA at the Games. Finally, Practitioner 4, 7, 8, 11, and 12 particularly emphasized the need to develop written VOA guidelines. The program is currently delivered, and facilitators are trained based on their experience. However, practitioners believe that a written set of guidelines is crucial for sustainable and consistent delivery of the VOA in the future. These suggestions highlight the practitioners' commitment to continually refining the VOA to maximize its impact on athletes and communities. By addressing the challenges and embracing opportunities for improvement, the VOA can continue to evolve and strengthen its role in empowering athletes and fostering positive change in the Pacific Islands and beyond.

## 4 Discussion

Practitioners' perspectives on the VOA offer critical insights into current practices and have significant implications for the direction of its future implementation. The central message of “empowering athletes” was highlighted in relation to the program's overarching aim. The opportunity for learning, which raises awareness of contemporary social issues and emphasizes the actions athletes can undertake as role models, was highly valued, constituting a crucial aspect of the VOA (32, 47). The VOA's potential for wider impact lies in its ability to inspire and empower athletes, who can then positively influence their communities and create a ripple effect of change and growth. Sport has the unique ability to unite people and transcend cultural, social, and economic barriers (48). The VOA leverages this power to educate athletes on essential topics and promote social change. By providing education and support across various aspects of athlete development and responsibility, the VOA inspires athletes to become positive role models and leaders. These athletes, in turn, have the potential to drive change and make a significant impact on their communities, promoting values such as integrity, health, environmental responsibility, and safety. The findings in this study also demonstrate the great potential of VOA Champions serving as practitioners within the program. The majority of practitioners in this study had personally participated in the VOA as athletes, and they provided insights into how the VOA positively influenced their careers and lives, ultimately fostering their development as role models, leaders, and empowered individuals. This holistic approach to athlete support not only enhances their performance as both athletes and practitioners but also contributes to their overall development and well-being (15, 16).

Practitioners appreciated the VOA's role in enabling athletes to develop transferrable skills for their career development and life after sport (49). By equipping athletes with essential life skills and transferrable competencies, the VOA supports their overall well-being and success beyond their sporting careers, ensuring a smoother transition into their post-sport lives. Given the significance of pre-retirement planning (19) and transferable skills (49) for athletes' life after sport, and the limited availability of athlete support programs in the Pacific Islands, it is critical to expand the VOA's implementation. In addition, incorporating more content to assist athletes with their transition out of sport could enhance the program's effectiveness and support (8, 11). Researchers have argued that support for athletes' career development and transitions should be provided to help them manage challenges and barriers they may face during and after their athletic careers (15, 19, 41). The importance of organizational support from sports organizations and national governing bodies has also been emphasized (8, 11, 50, 51). In this context, the findings of this study demonstrate that the ONOC has played a significant role in supporting athletes' career development and transitions.

Even though the VOA has shown positive outcomes, practitioners highlighted the need to further promote the program among athletes and communities in the Pacific Islands. This would enable more young individuals to benefit from these best practices. To achieve this goal, consideration should be given to increasing funding and budget allocations for the program, which may require attention from sport governing bodies such as the ONOC and IOC. Increased funding, partnerships, and accessibility can be critical to maximize the impact of the VOA and similar programs. Sufficient funding and resources can ensure the effective implementation and expansion of these programs, while partnerships with sports organizations, educational institutions, and community stakeholders can further enhance their reach and effectiveness (1). The practitioners also suggested that the VOA as a framework could be adapted to different cultural contexts, representing an excellent case of transferring best practices from the Global South to the Global North. This finding holds considerable importance, as existing literature on athlete support has predominantly concentrated on Western countries (19, 30). In order to adapt the VOA's practices for the Global North and other countries, the practitioners responsible for coordinating and leading the VOA highlighted the necessity of creating written guidelines that would facilitate the program's long-term implementation and success. In addition, to optimize the VOA delivery and experience, several suggestions were made by the practitioners including incorporating more interactive activities that engage athletes, utilizing larger spaces to accommodate more participants, promoting the VOA to reach a wider group of athletes. By implementing these improvements, the VOA can better cater to athletes' needs and enhance their overall experience and development.

In terms of increasing accessibility, it will enable a broader range of athletes to benefit from the VOA and contribute to their success. However, expanding the VOA's reach and impact in different regions presents both challenges and opportunities. Challenges include the need to adapt the program to diverse cultural contexts, limited resources, and varying levels of sport infrastructure as proved in this study. Despite such challenges and limitations, as the practitioners demonstrated, the VOA framework holds potential for cross-cultural learning and adaptability in diverse contexts. By understanding and accommodating cultural nuances (28), the VOA can effectively serve athletes from various backgrounds and address their unique needs. Cross-cultural learning, in turn, can contribute to the ongoing improvement and refinement of the VOA framework, ensuring its continued relevance and effectiveness in supporting athletes from around the world.

Communities of Practice (CoPs) are social learning processes within groups that share common interests, goals, and practices (33). The practitioners' approach in this study, during the delivery of the VOA, appeared to align with the CoP concept. Analyzing the VOA from the CoP perspective enabled the exploration of shared thoughts and experiences among practitioners, as well as the identification of positive outcomes and challenges in the program's delivery. This approach offers a deeper understanding of factors influencing practitioners' perceptions and experiences. Meanwhile, the CoP framework assists in identifying areas for improvement in VOA practitioners' delivery, ultimately contributing to athletes' personal and professional growth. By exploring the dynamics of CoPs within the VOA, this study offers valuable insights for sport governing bodies and other stakeholders involved in athlete support initiatives. These insights can inform the design and implementation of comparable programs in other regions and guide the development of strategies to enhance existing programs while promoting a culture of continuous learning and improvement among practitioners. It is hoped that the findings in this study can contribute to the ongoing development and effectiveness of the VOA and similar athlete support initiatives across diverse contexts.

## 5 Conclusions

The findings in this study make significant contributions to both academic literature and practical applications by providing insights from practitioners who deliver athlete support programs in the under-researched Pacific Islands region. These insights facilitate the identification of critical recommendations for developing effective strategies and initiatives to better support athletes in developing countries. First of all, we advocate for the continued emphasis on athlete empowerment within the program. To enhance this, it is critical to motivate athletes to engage with the VOA, helping them become knowledgeable about social issues and how sports, along with athletes as role models, can contribute to positive changes in society. Such engagement promotes athletes to leadership roles, further strengthening their empowerment. Thus, increased attention and investment are necessary to expand the program and reach more athletes in the region. This expansion will allow more athletes to benefit from the VOA, enabling them to influence young people as role models and leaders in their communities. Given the VOA's positive impact on athletes' preparation for life after sports, this content should be more clearly incorporated into the program, possibly as a new theme, with development based on relevant research evidence and regional athletes' experiences. For instance, career assistance programs (CAPs) highlighted in sports literature [e.g., (11, 18)], can help develop themes and content specifically designed to support athletes in preparing for their lives after sports. Developing guidelines may be crucial and even urgent for the program's continued growth and sustainability. Regarding the sharing of best practices from the Global South to the Global North, further research is encouraged to apply the VOA in countries in the Global North, with a focus on adapting the program to different cultural contexts. In addition, the present study employs the Communities of Practice (CoP) theoretical framework, demonstrating its applicability within the context of athlete assistance programs and initiatives. By examining the interactions and shared learning among practitioners, the study demonstrates the value of the CoP framework in understanding and enhancing the delivery of the VOA. As a result, this research not only fills a gap in the existing literature but also offers practical guidance for stakeholders looking to improve athlete support initiatives in diverse settings.

This study, while offering valuable insights, is subject to some limitations. Although it provides an in-depth exploration of practitioners' perspectives on the VOA and its delivery, it is crucial to explore athletes' perspectives on the program as well. While the larger project that this study is a part of addresses athletes' perspectives to some extent, obtaining more detailed insights from athletes could help further enhance and better facilitate the program. If it is feasible, conducting focus groups with practitioners to discuss athlete support programs could potentially strengthen the analysis of their perceptions and experiences from the Communities of Practice (CoP) perspective. In addition, it would be valuable to compare athletes' and practitioners' points of view, as there might be discrepancies in their perceptions that could be addressed to improve the program. Future research could benefit from incorporating these additional perspectives and employing varied research methodologies to provide a more comprehensive understanding of athlete support programs, such as the VOA. By doing so, researchers can continue to identify ways to optimize program delivery and better support athletes in their personal and professional development.

## Data Availability

The original contributions presented in the study are included in the article/Supplementary Material, further inquiries can be directed to the corresponding author.
